# Genetic linkage of distinct adaptive traits in sympatrically speciating crater lake cichlid fish

**DOI:** 10.1038/ncomms12736

**Published:** 2016-09-06

**Authors:** Carmelo Fruciano, Paolo Franchini, Viera Kovacova, Kathryn R. Elmer, Frederico Henning, Axel Meyer

**Affiliations:** 1 Lehrstuhl für Zoologie and Evolutionsbiologie, Department of Biology, University of Konstanz, Universitätsstrasse 10, 78457 Konstanz, Germany; 2 School of Earth, Environmental and Biological Sciences, Queensland University of Technology, Brisbane, Queensland 4000, Australia; 3 Department for Plant Developmental Genetics, Institute of Biophysics, Academy of Sciences Czech Republic, Královopolská 135, 612 65 Brno, Czech Republic; 4 Institute of Biodiversity, Animal Health and Comparative Medicine, College of Medical, Veterinary and Life Sciences University of Glasgow, Glasgow G12 8QQ, UK

## Abstract

Our understanding of how biological diversity arises is limited, especially in the case of speciation in the face of gene flow. Here we investigate the genomic basis of adaptive traits, focusing on a sympatrically diverging species pair of crater lake cichlid fishes. We identify the main quantitative trait loci (QTL) for two eco-morphological traits: body shape and pharyngeal jaw morphology. These traits diverge in parallel between benthic and limnetic species in the repeated adaptive radiations of this and other fish lineages. Remarkably, a single chromosomal region contains the highest effect size QTL for both traits. Transcriptomic data show that the QTL regions contain genes putatively under selection. Independent population genomic data corroborate QTL regions as areas of high differentiation between the sympatric sister species. Our results provide empirical support for current theoretical models that emphasize the importance of genetic linkage and pleiotropy in facilitating rapid divergence in sympatry.

## Abstract (web summary)

Genetic linkage of ecologically relevant traits has been suggested to facilitate sympatric speciation. Here, Fruciano *et al*. show in sister species of cichlid fish the genetic non-independence of genomic regions responsible for differentiation in body size and pharyngeal jaw morphology, two characters associated with adaptive divergence in sympatry.

## Graphical abstract

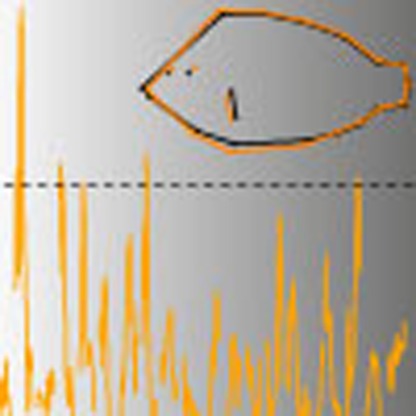

Sympatric speciation—the process by which new species arise in a geographic setting without barriers to gene flow—has been hotly debated over the past 50 years^1^,^2^. It has been controversial because of the restrictive genetic and environmental conditions probably needed for divergent selection to overcome the homogenizing effects of gene flow and produce different species in the absence of extrinsic barriers^2^,^3^,^4^. To date, only a small number of empirical examples are accepted to have fulfilled the conditions for speciation with gene flow: divergent host races in herbivorous insects^5^, divergence in palms and other plants promoted by diversity in soil on remote oceanic islands^6^ and trophically polymorphic crater lake cichlid fishes^7^,^8^. Consequently, the research focus has shifted to understanding the conditions that permit or promote sympatric speciation^2^,^4^,^9^. Most models of speciation, whether mathematical or verbal, require a strong role of close linkage^2^,^10^ of the genetic loci that underlie the diverging phenotypic traits. These include ‘divergence hitchhiking' models, in which a decrease of gene flow between populations in genomic regions surrounding loci under divergent selection can generate larger regions of differentiation between the diverging genomes^11^. Empirical tests of theoretical models on the few substantiated cases of sympatric speciation are, however, still scarce (but see refs 5, 12).

Nicaraguan crater lake cichlid fishes are one of those few well-substantiated instances of sympatric speciation^3^,^7^,^8^. The chain of crater lakes in Nicaragua has been independently colonized from the large and shallow Nicaraguan great lakes. Crater Lake Apoyo is maximally *ca*. 22,000 years old, small, deep and characterized by clear water^13^. A small and monophyletic adaptive radiation of six endemic cichlid species (*Amphilophus* spp. complex, or Midas cichlids) has formed rapidly^8^,^13^ and sympatrically into open water (limnetic) and bottom-dwelling (benthic) species^7^. These species differ in body shape (limnetics are more elongate)^13^,^14^ and trophic ecology^8^, have different gut bacterial communities^15^ and differ in the morphology of their lower pharyngeal jaws^7^ (modified gill arches that form a functional second jaw and are used to crush hard food; these are more robust in benthic forms; Fig. 1). Body shape has important ecological consequences and a genetic basis, as the difference between species is retained when fish are grown under common laboratory conditions^14^. The adaptive significance of variation in body shape has been shown directly in sticklebacks^16^ and perch^17^, where deep bodied fish perform better in benthic environments and more elongated fish perform better in the open water. Biomechanical studies of fish locomotion also suggest that a deeper body performs better when maneuvering in the more structurally complex benthic zone^18^. The adaptive significance of variation in pharyngeal jaw morphology in Midas cichlids has been evidenced experimentally. Indeed, the more robust pharyngeal jaws typical of the benthic forms perform better when processing hard food as compared with softer food items, whereas the opposite is true for the more gracile pharyngeal jaws of limnetic species^19^. The sympatric speciation and the well-studied traits involved in ecological divergence make the Midas cichlid species flock an ideal system to clarify the genetic basis of ecologically relevant traits and how selective pressures can translate into important genomic differences in the face of ongoing gene flow.

Theory predicts that linkage and/or pleiotropy might increase the likelihood or even facilitate sympatric speciation more generally, but especially as it applies to Midas cichlids^20^,^21^,^22^. Here we show that the quantitative trait locus (QTL) of largest effect for body shape and pharyngeal jaw shape overlap in a single linkage group (LG), making these two key ecological traits genetically non-independent. We also identify the co-localization of genes under selection and QTL for these traits. Our results, then, provide empirical support for current theoretical models of speciation with gene flow.

## Results

### Mapping of QTL

**Figure 2:**
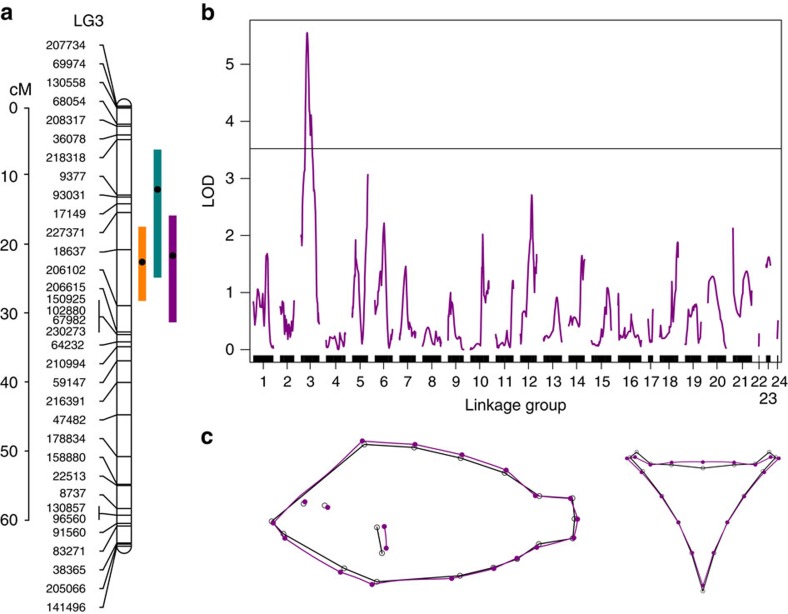
QTL mapping. (**a**) Bayesian credibility intervals for QTL in LG 3: orange bars for body shape; green bars for pharyngeal jaw shape; violet bar for covariation of body shape and pharyngeal jaw shape. (**b**) LOD scores at each position in each LG obtained by mapping PLS scores (covariation). The horizontal line identifies the genome-wide significance threshold obtained through permutations (at LOD=3.52). (**c**) Covariation accounted for by the only QTL (on LG 3) with LOD score higher than the genome-wide significance threshold.

To identify the genomic regions underlying ecologically relevant morphology, we performed QTL mapping on an interspecies genetic cross of benthic *Amphilophus astorquii* and limnetic *Amphilophus zaliosus.* From high-coverage sequencing of double-digest restriction site-associated DNA (ddRAD), we constructed a genetic map of 495 single-nucleotide polymorphisms (SNPs) resolving 24 LGs with an average marker spacing of 2.65 cM. This map was then used for multivariate QTL mapping of body shape and pharyngeal jaw size and shape. We found highly supported QTL for both shape traits (five QTL for body shape and three for pharyngeal jaw shape; see Supplementary Table 1 and Supplementary Figs 1 and 2 for location, confidence interval, effect size and predicted shape change of each QTL). We did not find any significant QTL for lower pharyngeal jaw size. Notably, the QTL of strongest effect for both body shape and pharyngeal jaw shape co-located on LG 3 and their confidence intervals overlap (Bayesian credibility intervals 18–28 cM for body, 6–24 cM for lower pharyngeal jaw shape; Fig. 2). This genetic non-independence is further supported by the analysis showing significant covariation (Escoufier RV=0.033, *P*=0.044) in body and pharyngeal jaw shape in the QTL mapping population of F_2_ individuals. When mapping covariation of body and pharyngeal jaw shape, a single highly supported QTL on LG 3 was found (Bayesian credibility interval 16–31 cM; Fig. 2). Finally, a statistical test based on permutations shows (overlapPermTest function of the regioneR package^23^, overlap=1, *P*=0.044) that the overlap of the QTL regions for body and pharyngeal jaw shape is higher than expected by chance. These results suggest a pleiotropic effect or a close linkage between the genetic loci underlying quantitative variation in these two ecomorphological traits.

### Co-localization of genes under selection and QTL regions

To identify what genes are potentially contributing to the benthic–limnetic divergence between species in Lake Apoyo by responding to positive selection, and whether they co-localize with QTL regions, we sequenced a Midas reference transcriptome combining the reads of five closely related species of the *Amphilophus* complex by Illumina next-generation sequencing. After merging a reference-guided and a *de novo* assembly, and retaining the transcript with the highest similarity score among those matching the same Nile tilapia protein, we obtained a Midas reference transcriptome of 15,348 sequences (N50 value: 5,067). After mapping the reads of our two focal species (*A. astorquii* and *A. zaliosus*) and extracting the species consensus (see Methods), we obtained a total of 11,103 genes orthologous between species, of which 71 showed signatures of positive selection (dN/dS >1; Supplementary Data 1). These genes had no significant over-representation of functional categories when compared against the full transcriptomic data set of orthologous genes, as revealed by enrichment analyses (Fisher's exact test; *P*>0.05). Mapping these 71 genes showing signatures of positive selection and all the linkage map markers on the Midas cichlid draft genome^8^, we found that three genes showing positive selection were associated with two QTL regions for the mapped phenotypic traits. One of these genes (*apolipoprotein eb-like*) co-located in a QTL region (in LG9) for body shape, whereas two genes (*cell cycle control protein 50a-like* and *sodium-coupled neutral amino acid transporter 4-like isoform x6*) clustered with the QTL for pharyngeal jaw shape on LG12 (see Supplementary Data 1 for details).

### Genetic divergence in QTL regions

**Figure 3:**
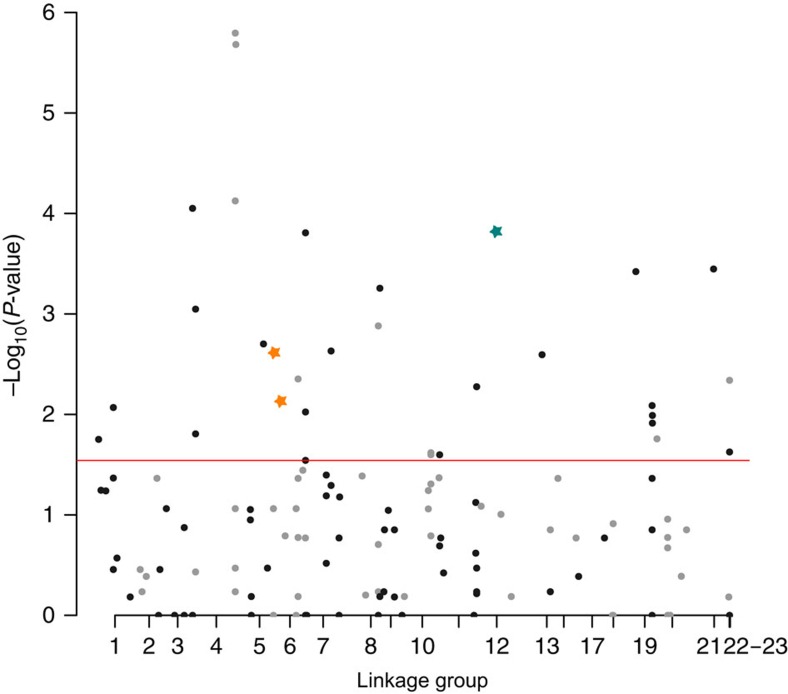
Population genomics. Manhattan plot of genome-wide differentiation between sympatric Midas species (significance levels for the Fisher test), including regions that co-localize with QTL. Only genomic windows containing RAD markers in the linkage map are plotted. The horizontal line represents a significance threshold obtained from the multiple test correction procedure (−Log_10_ (*P*-value)=1.49). Coloured stars represent genomic windows significantly differentiated between species and located in QTL regions (orange for body shape, green for pharyngeal jaw shape). SNPs not located in QTL regions are represented in grey or black, alternating these two colors so that the LGs are distinguishable.

To test whether the QTL regions co-localize with regions of genetic divergence between species in natural populations, we used pooled population genomic data of wild-caught individuals^8^. Although the average genome-wide differentiation between species was relatively low (average F_ST_=0.083 based on a 500 bp sliding window; Supplementary Data 2), the regions of the Midas cichlid genome unequivocally attributable to QTL regions (see Methods) were more differentiated between species (average F_ST_=0.097) than were the genomic regions containing non-QTL linkage map markers (average F_ST_=0.081, similar to the genome-wide F_ST_ computed using all data). Three genomic regions containing markers in the QTL intervals also show significant differences between species in allele frequencies (Fisher's exact test *P*<0.01 after correcting for multiple tests; Fig. 3 and Supplementary Data 3).

## Discussion

We have identified significant signals of positive selection and genomic co-localization of QTL underlying two major ecologically relevant traits in sympatrically speciating sister species of cichlids. Specifically, in cichlid fish from Lake Apoyo we identified five QTL for body shape and three for pharyngeal jaw shape. However, we did not identify any QTL for pharyngeal jaw size. This, combined with the fact that there is no significant difference in pharyngeal jaw size between the two species when kept under captive conditions, suggests that the variation in pharyngeal jaw size between these species is perhaps more affected by the environment. Indeed, although the existence of pharyngeal jaws and their diversification have been implicated in playing a causal role in the spectacular diversification of cichlids, these structures are well known to exhibit phenotypically plastic variation depending on the hardness of consumed food items^24^. The QTL for body shape show diverse effects on this trait (Supplementary Fig. 1c). It is remarkable that the QTL of strongest effect for body and pharyngeal jaw shape, overlapping in LG3, produce, respectively, a deepening of the middle of the body and an enlargement of the posterior margin of the pharyngeal jaw. These shape changes match the variation between species previously described in natural populations^7^,^13^ and have a clear functional relevance. Indeed, a deeper body is advantageous for swimming in a benthic environment^18^, where an enlarged pharyngeal jaw is beneficial^19^ because of the higher abundance of hard prey. The genomic regions containing QTL for body and pharyngeal jaw shape also contain expressed genes that show signals of response to positive selection and their function has been linked to growth and cell cycle control^25^. For this reason, these three genes are promising candidates that might contribute to the observed morphological diversification between the two focal species. However, experimental validation is necessary to clarify their causal role.

Further, the regions containing QTL showed significantly different allele frequencies between species, thus giving a clear genomic signal of divergent natural selection acting on these traits or at tightly linked genes.

This finding is extremely important as current models of speciation emphasize the importance of tight linkage and pleiotropy in rapid speciation^26^, yet, so far, there is a lack of empirical examples supporting them. Recent simulation studies^21^ show that the arrangement of genes in genomes is important to facilitate divergence. In particular, not only is the statistical association among large numbers of genes important for rapid divergence, but so also is divergent selection on persistent allelic combinations among these genes^22^. These genomic associations are important factors, especially in sympatry, and can alleviate the need for assortative mating^21^. The exact role of tight linkage and pleiotropy in rapid speciation is still debated—as they can both conceivably favour and hinder divergence^26^. In the presence of very strong divergent selection, linkage might not contribute substantially to the establishment of divergently selected alleles (that is, they would diverge anyway whether linked or not, whereas with milder divergent selection linkage might be more important in favouring divergence). Our data, however, does not support this scenario in Midas cichlids. In fact, we would expect that very strong divergent selection on a few loci would result in a strong divergence in surrounding regions, which would be reflected in much higher F_ST_ estimates in QTL regions compared with the rest of the genome. In our case, instead, F_ST_ estimates in the QTL regions are just slightly higher than in the rest of the genome, suggesting not very strong divergent selection, a condition in which linkage could play a substantial role in promoting sympatric divergence.

The most recent theoretical predictions based on simulations^21^ suggest that pleiotropy or tight linkage are particularly—but not exclusively—important in favouring divergence in conditions of many genes under selection, genes of small effect and high gene flow. The number of genes under selection is important, because genomes tend to become more consolidated if many loci are contributing to divergence^21^. Clearly, in our case an accurate estimate of the number of genes underlying adaptive traits and under divergent selection is not possible due to empirical detection limits, such as the difficulty of detecting small-effect QTL. However, our empirical results generally conform to these recent theoretical expectations, as we find 71 candidate genes under selection and eight QTL regions for two traits. It is also likely to be that we failed to detect a number of smaller effect QTL, and that some of these might be genetically non-independent across the two traits.

High gene flow is supported in Apoyo Midas cichlids by the low average F_ST_, which is concordant with previous estimates on the same system based on different genetic markers^7^,^27^. Similar F_ST_ estimates have been documented in situations of divergence with gene flow in sticklebacks^28^. Low values of F_ST_ are probably also due to recent divergence. Indeed, the values of F_ST_ we observe are of similar magnitude to the ones observed in populations of another cichlid fish, *Archocentrus centrarchus*^29^, which recently diverged in allopatry in Nicaraguan lakes.

The last condition under which pleiotropy or linkage are expected to substantially favour divergence—the condition of genes of small effect—seems also to be satisfied in our study system. Effect sizes are not easily comparable across studies, as the inherent bias in their estimation^30^ might vary in extent depending on how a trait is defined and how it is mapped. However, other QTL studies on body shape have reported much higher effect sizes (typically around 10% in at least one QTL, sometimes as high as 20–30%) in a range of fish species^31^,^32^. In our study, instead, the QTL of largest genetic additive effect for any of the two traits accounts for a mere 4.08% of trait variance. Then, the effect sizes for QTL of body and pharyngeal jaw shape that we found in this study seem also to satisfy the condition of genes of small effect.

Thus, by largely matching theoretical predictions, our empirical results overall suggest that pleiotropy or tight linkage of QTL for different traits can facilitate rapid sympatric divergence.

## Methods

### Data sets used

For QTL mapping, a total of 305 F_2_ individuals from an *A. astorquii* × *A. zaliosus* cross were used in the present study. Briefly, a wild-caught female *A. astorquii* was crossed with a wild-caught male *A. zaliosus* and the eggs were removed from the parental tank once spawned. On maturity (*ca*. 1 year of age), we randomly chose one pair (F_1_) as it formed, isolated them into a different tank and allowed them to breed. This F_1_ pair produced the F_2_ individuals used here for QTL mapping. All fish were photographed in a standardized manner for morphometric analyses of body shape at 18 months of age. Further details on the cross are provided in ref. 14, where we also show that the two parental species retain differences in body shape even when raised under the same laboratory conditions. We performed a preliminary exploratory analysis of pharyngeal jaw size and shape on a small set (five *A. astorquii* and ten *A. zaliosus*) of wild-caught lab-reared individuals of each parental species. These showed nonsignificant difference in pharyngeal jaw size (analysis of covariance using body centroid size as a covariate for allometric correction; *P*=0.32) but significant difference in average pharyngeal jaw shape (Procrustes distance 0.042; *P*=0.0014 based on 10,000 permutations). The individuals used here include the ones analysed in ref. 14 (according to German law on animal welfare and specifically approved by the Regierungspräsdium Freiburg, Abteilung Landwirtschaft, Ländlicher Raum, Veterinär- und Lebensmittelwesen; approval G-11/ 73 35-9185.81), complemented by new specimens, which had not been sequenced before. Individuals of the F_2_ mapping population were tagged and later killed to dissect the lower pharyngeal jaw (*n*=265 due to part of the mapping population losing their transponder tag) at two time points. At this stage, we also took a second picture of their body to compute body centroid size and to analyse covariation between body and pharyngeal jaw shapes. To quantify the morphology of body and pharyngeal jaws, we used geometric morphometrics and analysed the set of points depicted in Supplementary Fig. 3. As in our previous study^14^, configurations of points were aligned through a generalized Procrustes analysis with sliding of semi-landmarks^33^ and allometric variation was removed from the data using, for both body and pharyngeal jaw shape, residuals of regression on body centroid size. In the case of body shape, before this regression, we also carried out a procedure for removal of body arching^34^,^35^,^36^. For pharyngeal jaws, we performed the analysis of shape only on the symmetric component^37^, as this is the ecologically relevant component of shape variation that distinguish the two species (that is, enlarged pharyngeal jaws have been described in benthic Midas cichlids, as opposed to the more gracile pharyngeal jaw of the open water species). We used, as measures of pharyngeal jaw size, pharyngeal jaw centroid size and pharyngeal jaw weight. Each of these measures of pharyngeal jaw size was regressed on body centroid size and the residuals were used in subsequent analyses so as to account for allometric variation. We chose to use two different measures of pharyngeal jaw size, because they capture different aspects of pharyngeal jaw morphology: centroid size captures change in overall size, whereas weight is also affected by changes in bone density. For both the analyses of pharyngeal jaw size and shape, we removed the variation between time points before downstream analyses.

To analyse covariation between body and pharyngeal jaw shape, we used the Escoufier RV coefficient^38^ and partial least squares analysis (PLS)^39^ on the allometry-corrected shape variables. The Escoufier RV coefficient was used as a multivariate measure of association to test the null hypothesis of complete independence between body and pharyngeal jaw shape using the permutational procedure implemented in MorphoJ v1.06d^40^. If body and pharyngeal jaw shape were genetically independent, we would expect this statistical test to be nonsignificant in the F_2_ mapping population. PLS was used to identify directions of maximal covariation between body and pharyngeal jaw shape. Only the first pair of axes—one for body, the other for pharyngeal jaw shape—was significant. We then used scores of each individual along each of these PLS axes for QTL mapping of body–pharyngeal jaw shape covariation.

A second set of individuals from each parental species, plus other three species of the Midas group, was used in the transcriptomic-based analysis to detect genes under selection. Although sequence evolution was analysed only in the two focal species, the other three species were included to generate a high-quality Midas reference transcriptome^41^. Two broods each from five Midas species were produced and sampled at 1 day post hatch (1 dph) and 1 month post hatch (1 mph): *A. astorquii* and *A. zaliosus* (crater Lake Apoyo), *A. amarillo* and *A. sagittae* (crater Lake Xiloá), and *A. citrinellus* (Lake Nicaragua). In total, nine samples per species were used for RNA extraction and RNA sequencing: three for the 1 dph stage and six for the 1 mph stage. Each of the 1 dph samples was obtained pooling three individual fish. The 1 mph samples were obtained using three fish and separating the head and the rest of the body (that is, head+body × 3 individuals=6 samples). Individuals from the two different broods were included in the 1 dph and 1 mph samples.

For the population genomic analyses, we used a published data set^8^ consisting of whole genome sequences of 26 pooled wild-caught individuals (PoolSeq) for each of *A. astorquii* and *A. zaliosus*.

### Molecular methods and genotyping

Genomic DNA was extracted from the fin tissue of the two parentals, the two F_1_ and 305 F_2_ individuals using the Qiagen DNeasy Blood & Tissue Kit (Qiagen, Valencia, USA) following the manufacturer's protocol. The DNA quality of each sample was determined by agarose gel electrophoresis and quantified using a Qubit v2.0 fluorometer (Life Technologies, Darmstadt, Germany). Approximately 300 ng of DNA template of each sample was used to construct ddRAD^42^ libraries following the modifications introduced in ref. 14. Seven ddRAD libraries, containing from 45 to 50 barcoded individuals each (see Supplementary Data 4 for details), were prepared and single-end sequenced in an Illumina HiSeq 2000 using four-colour DNA sequencing-by-synthesis technology with 101 cycles.

After barcode demultiplexing and filtering out low-quality reads with the ‘process-radtags' script implemented in the Stacks v1.20 pipeline^43^, a total of 8,748,063 (male) and 7,434,360 (female) sequences were obtained for the parents, 4,085,710 (male) and 3,799,752 (female) for the F_1_, and an average of 2,396,189 sequences for the F_2_ progeny (s.d. 984,832). Sequence length of each read was of 96 bp after removing its 5 bp barcode.

Genotyping was performed using the ‘denovo_map.pl' module of Stacks with the parameters described in ref. 14, except for the coverage threshold to export SNPs in the Stacks' ‘genotypes' script (-m) that was set at 10 and 15 in two different runs.

### Linkage map construction

Linkage map construction was performed using the programme JoinMap v4.0 (ref. 44), which calculates genetic linkage maps in experimental populations of diploid species. To infer genetic linkage, we used both the methods implemented in JoinMap (the regression-based algorithm that uses the Kosambi mapping function and the Monte Carlo maximum likelihood mapping algorithm). This allowed us to identify inconsistencies between methods. A first linkage map was estimated to serve as a reliable backbone using a data set exported from Stacks that had higher coverage threshold (–m15). To increase marker density, the orders obtained with this data set were given as fixed orders to estimate the LGs with a genotype data set exported from Stacks that had a slightly less stringent coverage threshold of –m10 and pre-mapping filtering of loci with extreme segregation distortion (SD) levels (*P*<0.005). High levels of missing observations and SD can disturb the grouping phase of linkage map estimation^45^.

To estimate a reliable backbone for adding more markers, markers with >20% missing data (> 61.6 missing genotypic observations) and under high levels of SD (*χ*^2^: *P*<0.010) were excluded before grouping markers into LGs. Grouping was carried out using an independence LOD (logarithm of the odds, to the base 10) threshold of >5. The order of the markers in each LG was estimated using the maximum likelihood mapping function in JoinMap. Genotypes were visually inspected for all LGs and anomalous loci (those with a high incidence of double recombinations) were excluded.

The –m10 data set had a total of 1,766 loci, which was reduced to 512 by eliminating markers with >35% missing data or extreme levels of SD that indicate genotyping errors (*P*>0.005)^46^. The final map was produced by (i) assigning the markers (*n*=410/512) with up to 20% missing data and *P*>0.01 to LGs using a independence LOD cutoff of 5 (as above); (ii) assigning markers with a higher level of missing observations (between 20% and 35%) and SD (0.01>*P*>0.005) to the groups based on the strongest- cross-link values; (iii) giving the fixed orders estimated with the stringent data set; and (iv) excluding anomalous loci after inspecting the visual genotypes, congruence of the maximum likelihood and regression algorithms, the number of recombinations, incidence of improbable genotypes and nearest-neighbour stress values^47^.

### QTL mapping

We separately mapped body and pharyngeal jaw shape and PLS scores (covariation) using the multivariate version of Haley–Knott regression^48^ on genotype probabilities implemented in the shapeQTL R package^49^. Body and pharyngeal jaw allometry-corrected shape variables were subjected to principal component analysis, to remove zero-variance dimensions before multivariate Haley–Knott regression. Using this multivariate approach, we could map all the variation in shape, as opposed to the projection on the between-group principal component we used previously^14^. Genotype probabilities were computed at 1 cM steps. A genome-wide significance LOD-score threshold was obtained using 1,000 random permutations under the null hypothesis of no association between the trait of interest and the genotype probabilities. For each QTL deemed significant at the 5% probability level, we estimated in shapeQTL the Bayesian credible interval for its position and its effect size in sum of squared deviations from the mean. Finally, we obtained predictions of QTL effect in terms of shape change vectors and PLS change estimates, which then we visualized in MorphoJ. Using a method to estimate statistical power in Haley–Knott regression^50^, our sample sizes (*n*=305 for body shape and *n*=265 for pharyngeal jaw shape) would result in an estimated statistical power of 0.98 (body shape) and 0.96 (pharyngeal jaw shape), to detect a QTL explaining 5% of heritable phenotypic variance, under a type I error rate of 0.05 and a marker distance of 2.65 cM (that is, the average marker spacing in our map).

Finally, we used a recently developed approach^23^ to test for the overlap of QTL regions for different traits. Given two sets of genomic regions and knowledge of the size of the LG, this approach uses random permutations to generate an empirical distribution of the number of overlaps between the two sets of genomic regions. The observed number of overlaps is then compared with this empirical distribution to obtain a *P*-value.

### Transcriptomic analysis

FastPrep-24 homogenizer (MP Biomedicals) tubes were used to process 30 μg of each sample (30 s at 4.0 M). Total RNA from each sample (see paragraph ‘Data sets used' above for details) was isolated using a Qiagen RNeasy Mini Kit with 100% ethanol used in all wash steps. RNA quality and quantity was assessed using a Bioanalyzer 2100 and a Qubit 2.0 fluorometer, respectively. Five-hundred nanograms of high-quality RNA (RNA Integrity Number value >8) was used to construct a barcoded sequencing library for each of the nine samples per species using the Illumina TruSeq RNA sample preparation kit (Low-Throughput protocol) according to the manufacturer's instructions (Illumina, San Diego, USA). To increase the average library insert size, chemical fragmentation was performed at 94 °C for 1 min. Paired-end sequencing of clustered template DNA was performed in an Illumina HiSeq 2500 with 309 cycles (151 cycles for each paired-read and 7 cycles for the barcode sequences).

After sequencing and pooling the different samples for each of the five species, we obtained 490,293,234 raw reads (from 81,089,742 to 115,093,936 reads per species). Remaining adapters were removed and low-quality reads were filtered out using the software Trimmomatic v.0.32 (ref. 51) with default parameters and discarding sequences shorter than 50 bp. Reference-guided and *de novo* assembly approaches were performed using the filtered reads of the 45 samples combined. For reference guided assembly, we used the programme Stringtie v1.0.4 (ref. 52), setting the Midas genome as reference. TopHat v2.0.14 (ref. 53) and Bowtie2.2.3 (ref. 54) were used to map reads onto the Midas genome using default parameters. Samtools v1.2.1 (ref. 55) was used to convert the Bowtie output alignment from SAM to BAM format, to obtain the input file for Stringtie. The *gffread* utility implemented in the Cufflink v2.2.1 package^56^ was used to extract the transcripts from the Midas genome. For the *de novo* assembly, the software Trinity v2.06 (ref. 57) was used to assemble the Midas reads (PasaFly transcript reconstruction mode, k-mer size of 32 and a minimum contig length of 200 bp). The obtained *de novo* and reference-based assemblies were combined and subjected to similarity searches (BLASTx v2.2.26 (ref. 58)) against the Nile tilapia (*Oreochromis niloticus*) protein data set (Ensembl release 73) using *e*-value=1*e*^−10^ as cutoff. The longest Midas transcript among those matching a unique tilapia protein was selected and its coding region was extracted according to the BLAST hit coordinates using bedtools v2.25.0 (ref. 59).

To infer orthology between the transcripts of the two focal species (*A. astorquii* and *A. zaliosus*), we implemented the following workflow. First, the extracted Midas cichlid coding regions were used as reference to independently align reads from the two focal species with CLC Genomics Workbench v6.5.1 (CLC bio, Aarhus, Denmark) with default parameters. Second, the consensus sequence was extracted from each alignment by exporting heterozygous sites, sites with low sequencing depth (threshold 10 × ) and low quality sites as unknown nucleotides (Ns). Finally, orthologue sequences were aligned with ClustalW v2.1 (ref. 60) and a custom Bash script was used to filter out sequence pairs with less than 30 complete codons. By explicitly coding heterozygous nucleotide sites within species as Ns and by removing shorter sequences, this workflow reduces the chances of finding false-positive genes under selection due to sequencing errors and decreases the probability of assuming that SNPs are fixed between species when they may actually be polymorphic within them.

This data set of 11,103 orthologue sequence pairs (where the alignable coding sequence length of the pairs ranged from 90 to 11,037 bp) was analysed to detect signatures of positive selection using the dN/dS approach^61^ (the ratio between non-synonymous, dN, and synonymous, dS, substitutions). As estimation of dN and dS rates can be influenced by alignment methods, we used two different alignment methods (ClustalW^60^ and the Needleman–Wunsch algorithm^62^), and three different approaches for the estimation of dN and dS. In particular, orthologous sequences aligned with ClustalW were analysed using separately the methods of Nei and Gojobori^63^, and the Yang and Nielsen^64^ with the PAML package v4.7a (ref. 65). The sequences aligned using the Needleman–Wunsch algorithm^62^ with no penalty for sliding were analysed using the the method of Goldman and Yang^66^, as implemented in the Matlab Bioinformatics Toolbox (Mathworks, Inc.). Out of the 11,103 orthologue sequence pairs, 298 had at least one polymorphic site (synonymous or non-synonymous; notice that codons containing ambiguous bases were excluded from the analysis). To avoid comparing paralogous genes, sequence pairs for which dS >0.1 were filtered out. This reduced the data set to 291 sequences. Exploratory scatterplots did not reveal any correlation between analysed sequence length (in codons) and dN, dS or dN/dS ratio. We considered and retained only sequences where dN/dS was consistently >1 in all three methods described above as candidate genes for selection. Finally, to further reduce the chances of false positives, all orthologue sequence pairs with dN/dS >1 were manually inspected to identify potential alignment errors. In all, this analysis suggested the presence of 71 candidate genes for selection between the two focal species.

Blast2GO^67^ was used to perform the functional annotation of these 71 candidate genes. The same gene set was tested for overrepresentation of Gene Ontology terms by an enrichment analysis based on the Fisher's exact test (false discovery rate=0.05) as implemented in Blast2Go. To test for Gene Ontology over-representation of the candidate genes relative to background, we compared them with the 11,103 genes that mapped uniquely to the tilapia proteins

### Co-location of genes with dN/dS > 1 and QTL

The 49 RAD markers included in the QTL credibility intervals and the 71 candidate genes were aligned to the Midas draft genome v5 (ENA accession number ID PRJEB6974) using the BLASTn algorithm. For each query, the top BLAST hit was recorded and BLAST output parsed using a MySQL database. Given the length of the draft Midas genome scaffolds (maximum 8.1 Mb) and the average Bayesian confidence interval for the detected QTL (16.9 cM) and considering a genome size of 840 Mb, genes were considered as belonging to the QTL regions when both BLAST queries (RAD marker and gene under selection) were aligned to the same reference Midas scaffold.

### Population genomics

We used low-coverage whole genome sequences for two pools of 26 wild-caught individuals for each of the two focal species^8^. A total of 69,161,026 (*A. astorquii*) and 92,024,490 (*A. zaliosus*) raw 151 bp paired-end reads were quality controlled using Trimmomatic. The filtered reads were then aligned to the Midas genome v5 using Bowtie and the output mapping files (BAM format) were then processed with Picard-tools v1.119 (http://picard.sourceforge.net.), to remove duplicates. Low-quality alignments (reads with mapping quality lower than 20, unmapped reads, reads in which both mates failed to align to the reference genome) were filtered out using the SAMtools *view* module. SAMtools *mpileup* module was used to extract SNP and coverage information from each pool. The average genome-wide coverage for the two species pools combined before and after the filtering steps applied was of × 15.2 and × 9.2, respectively (the distribution is shown in Supplementary Fig. 4). The PoPoolation2 v1.20127 (ref. 68) pipeline was then used to compute the fixation index (F_ST_) and to perform a test of difference in allele frequencies (Fisher's exact test). More specifically, before computing these statistics, genomic sites were subsampled to the target coverage of ten, to avoid bias across sites produced by a non-uniform coverage (before subsampling, 48.5% of the Midas genome had a per-base coverage of at least 10 × —see Supplementary Fig. 4). The minimum minor allele count at each site was set to 4, to drop only very rare alleles and to avoid overestimating heterozygous positions. The above-mentioned statistics were computed on non-overlapping windows of 500 bp with a minimum covered fraction of 0.5 (that is, at least half of the window has a coverage of 10 × and is included in the analysis), because using a sliding window approach reduces stochastic errors^69^. With these settings, we obtained 5,469 polymorphic sites (SNPs) covered by at least 10 reads in each species pool, which rendered 627 windows for which F_ST_ values were calculated. The *P*-values obtained with the Fisher's test were corrected for multiple tests using the binomial sequential goodness of fit procedure^70^.

### Co-location of QTL and population genomic data

The genomic scaffolds containing the RAD markers in the linkage map identified with the procedure described above (Co-location of genes with dN/dS > 1 and QTL) were also used in the interpretation of the results of the population genomic analyses. We computed the average F_ST_ for genomic scaffolds containing RAD markers in the QTL regions and the average F_ST_ for the genomic scaffolds containing the remaining RAD markers in the linkage map. We used the same principle to identify whether any of the QTL regions displayed significantly different allele frequencies.

### Data availability

The population genomic data and the corresponding reference genome are available in the European Nucleotide Archive (accession numbers PRJEB6990 and PRJEB6974, respectively). The remaining data that support the findings of this study are available from the corresponding author upon request.

## Additional information

**How to cite this article**: Fruciano, C. *et al*. Genetic linkage of distinct adaptive traits in sympatrically speciating crater lake cichlid fish. *Nat. Commun.* 7:12736 doi: 10.1038/ncomms12736 (2016).

## Supplementary Material

Supplementary Information Supplementary Figures 1-4, Supplementary Table 1

Supplementary Data 1 Genes under selection. Annotation, position in the Midas draft genome and localization in the QTL Bayesian credibility intervals for each gene with dN/dS higher than 1 in all four procedures used (see Methods for details) are shown.

Supplementary Data 2 FST values. The values refer to genomic windows of 500 bp within the genomic scaffolds containing RAD markers in the map and, therefore, unequivocally attributable to linkage groups and QTL regions.

Supplementary Data 3 Significance of Fisher exact test. The values, computed on genomic windows of 500 bp within the genomic scaffolds containing RAD markers in the map, are provided both as raw p-values and as values corrected with the SGoF Binomial procedure. When multiple markers blasted on the same genomic scaffold, only one has been retained.

Supplementary Data 4 Sequencing statistics of the individuals used in the QTL analysis. For each parent, F1 and F2 individual, the number of retained reads after quality filtering and the RAD library in which it was included are shown. When multiple markers blasted on the same genomic scaffold, only one has been retained.

## Figures and Tables

**Figure 1:**
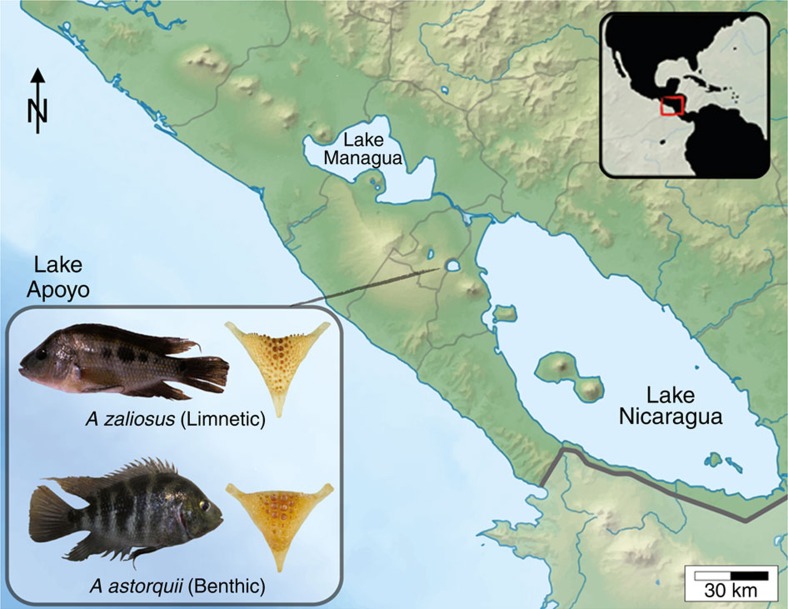
Nicaraguan lakes and benthic/limnetic Midas cichlids. The lake indicated is the crater lake Apoyo. In the inset, pictures of representative specimens of *A. astorquii* and *A. zaliosus*, and of typical lower pharyngeal jaws of these species.
