# Supplementary material for: Genetic linkage of distinct adaptive traits in sympatrically speciating crater lake cichlid fish
Source: Nat Commun. 2016 Sep 6;7:12736. doi: 10.1038/ncomms12736 (PMC5025864; doi:10.1038/ncomms12736)
Supplement: Supplementary Information — Supplementary Figures 1-4, Supplementary Table 1 [file ncomms12736-s1.pdf]

Supplementary Figures

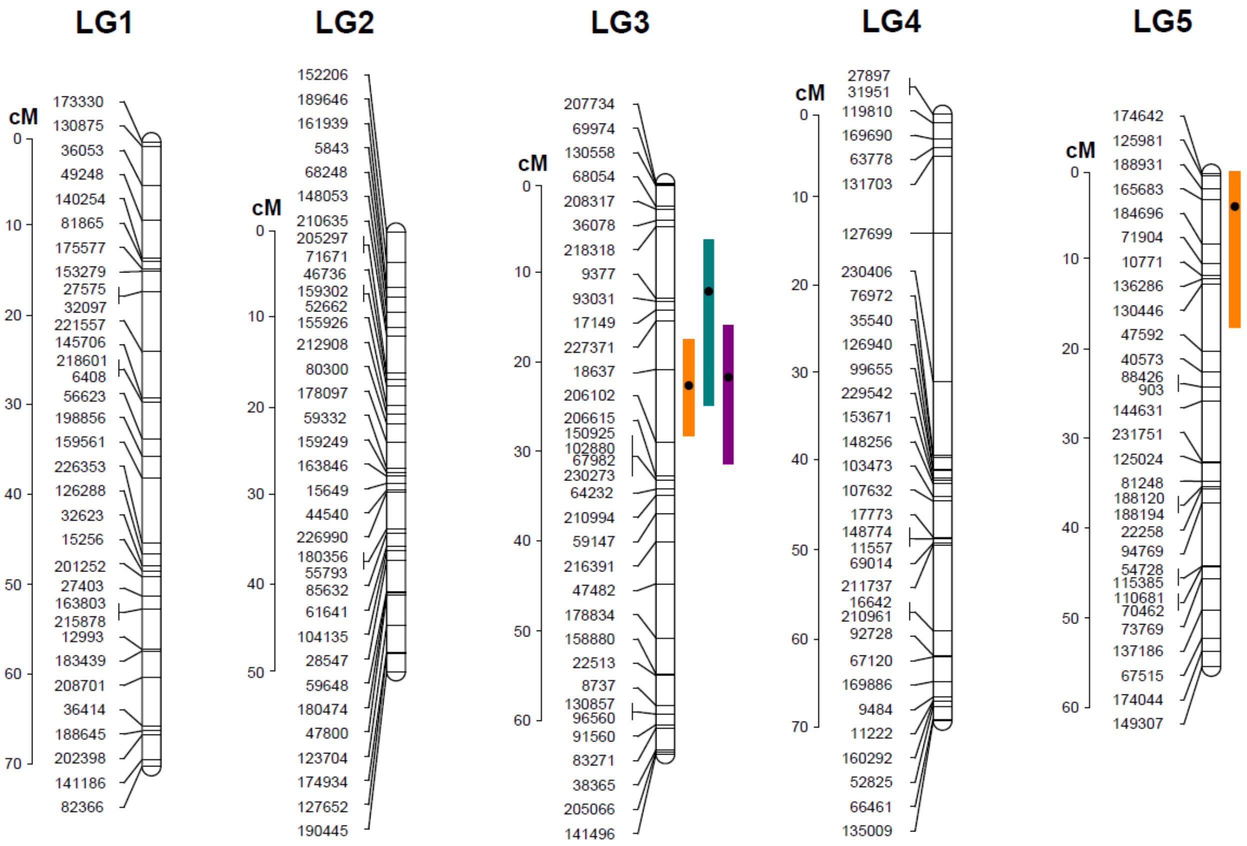

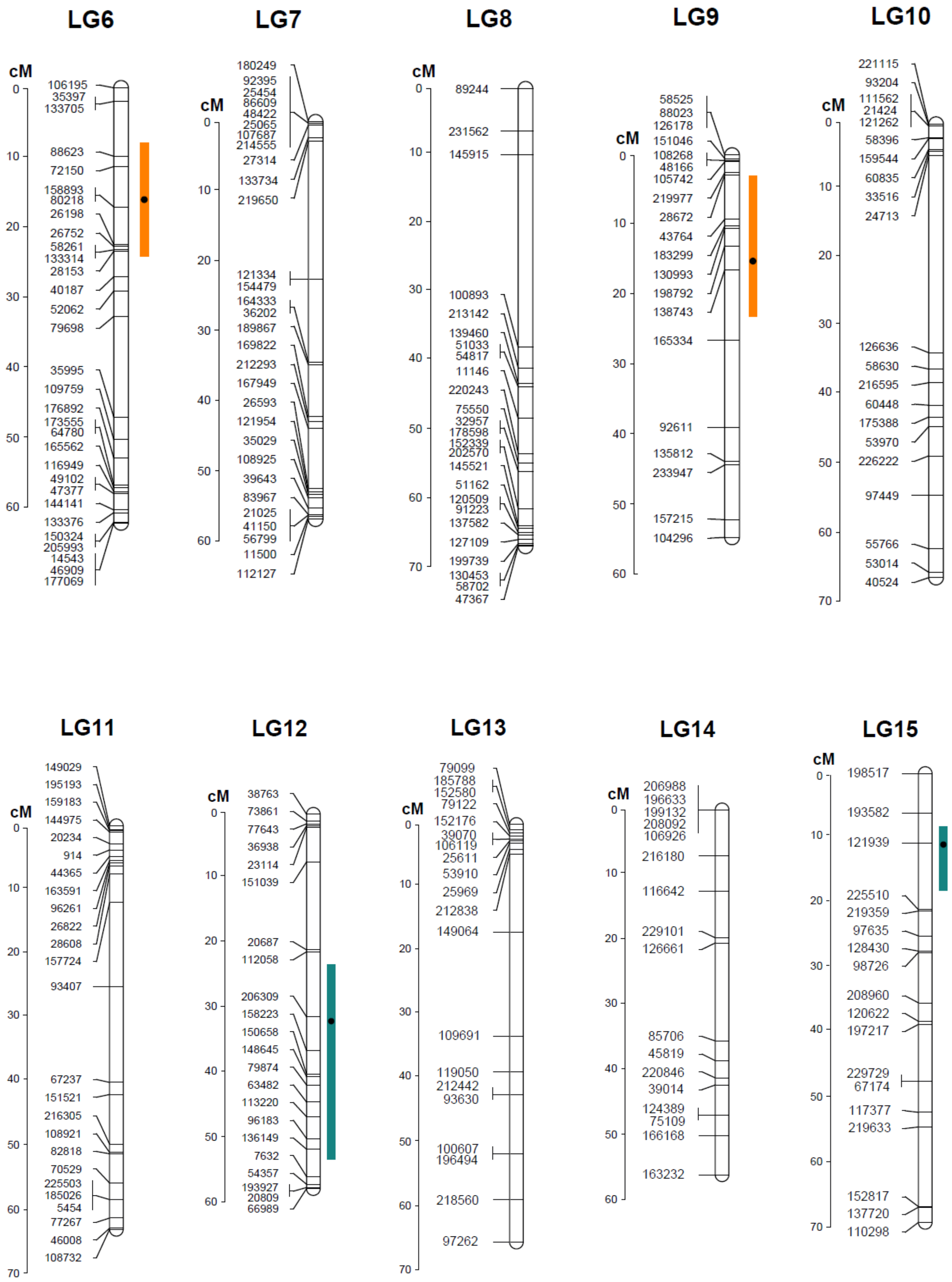
LG6
cM
0
10
20
30
40
50
60
106195
35397
133705
88623
72150
158893
80218
26198
26752
58261
133314
28153
40187
52062
79698
35995
109759
176892
173555
64780
165562
116949
49102
47377
144141
133376
150324
205993
14543
46909
177069
LG7
cM
0
10
20
30
40
50
60
180249
92395
25454
86609
48422
25065
107687
214555
27314
133734
219650
121334
154479
164333
36202
189867
169822
212293
167949
26593
121954
35029
108925
39643
83967
21025
41150
56799
11500
112127
LG8
cM
0
10
20
30
40
50
60
70
89244
231562
145915
100893
213142
139460
51033
54817
11146
220243
75550
32957
178598
152339
202570
145521
51162
120509
91223
137582
127109
199739
130453
58702
47367
LG9
cM
0
10
20
30
40
50
60
58525
88023
126178
151046
108268
48166
105742
219977
28672
43764
183299
130993
198792
138743
165334
92611
135812
233947
157215
104296
LG10
cM
0
10
20
30
40
50
60
70
221115
93204
111562
21424
121262
58396
159544
60835
33516
24713
126636
58630
216595
60448
175388
53970
226222
97449
55766
53014
40524
LG11
cM
0
10
20
30
40
50
60
70
149029
195193
159183
144975
20234
914
44365
163591
96261
26822
28608
157724
93407
67237
151521
216305
108921
82818
70529
225503
185026
5454
77267
46008
108732
LG12
cM
0
10
20
30
40
50
60
38763
73861
77643
36938
23114
151039
20687
112058
206309
158223
150658
148645
79874
63482
113220
96183
136149
7632
54357
193927
20809
66989
LG13
cM
0
10
20
30
40
50
60
70
79099
185788
152580
79122
152176
39070
106119
25611
53910
25969
212838
149064
109691
119050
212442
93630
100607
196494
218560
97262
LG14
cM
0
10
20
30
40
50
60
206988
196633
199132
208092
106926
216180
116642
229101
126661
85706
45819
220846
39014
124389
75109
166168
163232
LG15
cM
0
10
20
30
40
50
60
70
198517
193582
121939
225510
219359
97635
128430
98726
208960
120622
197217
229729
67174
117377
219633
152817
137720
110298

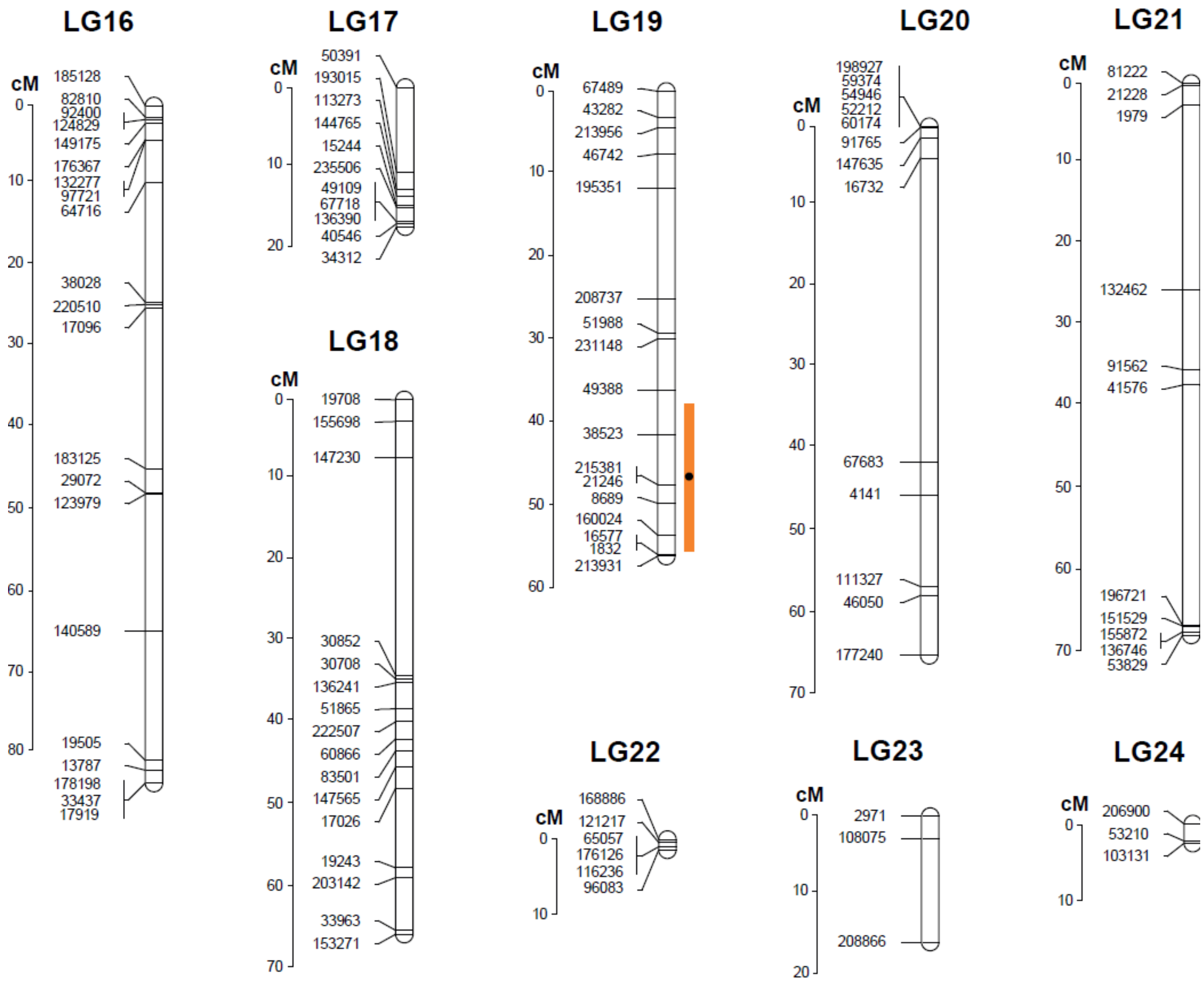

Supplementary Figure 1 | Linkage map and QTL location. Linkage groups (LG) obtained with an F2 mapping design (*A. astorquii* x *A. zaliosus*). Recombination map distances are indicated with a scale in centiMorgan (cM) on the left of each LG. Marker IDs, as numbered by the Stacks software, are given on the left of the LGs with lines pointing their position. Colored bars on the right of the LGs (when present) indicate the Bayesian confidence interval for the QTL position: orange bars, body shape; green bars, pharyngeal jaw shape; violet bar, covariation of body shape and pharyngeal jaw shape. Black dots on the colored bars indicate the position at which the highest LOD score of the QTL is found (see Supplementary Table 1 for details).

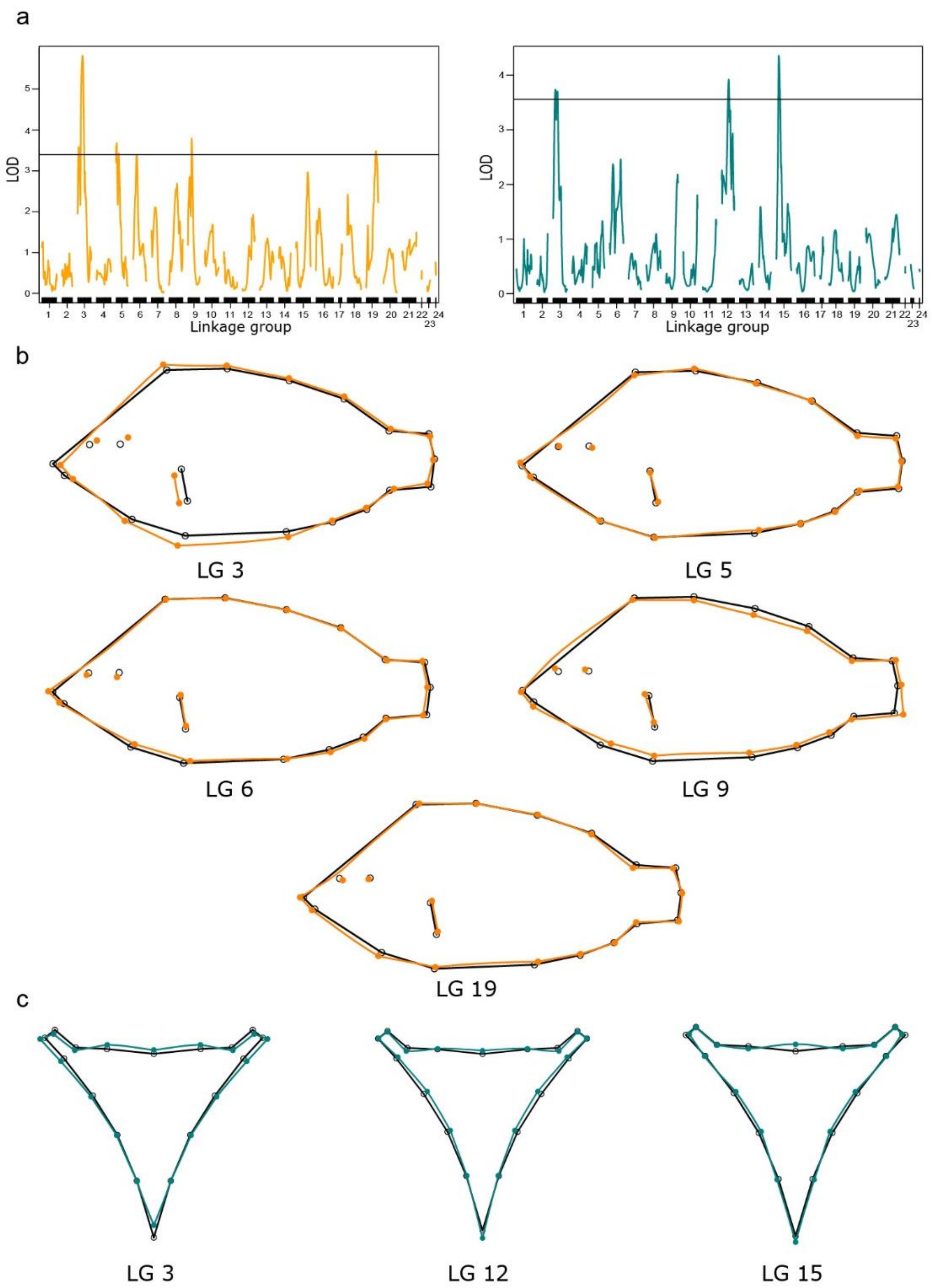

Supplementary Figure 2 | LOD score profiles and additive genetic effect of QTL. (**a**) LOD scores at each position in each linkage group (left, body shape; right, pharyngeal jaw shape). The horizontal line identifies the genome-wide significance threshold obtained through permutations

(LOD=3.39 for body shape, LOD=3.56 for pharyngeal jaw shape). (**b)** Additive genetic effect at each QTL for body shape. (**c**) Additive genetic effect at each QTL for pharyngeal jaw shape. Effects are depicted as shape change (colored wireframe) relative to a common starting shape (black wireframe). Within each trait, effects are scaled in the same way so that higher difference between the colored and the black wireframes correspond to higher effect sizes.

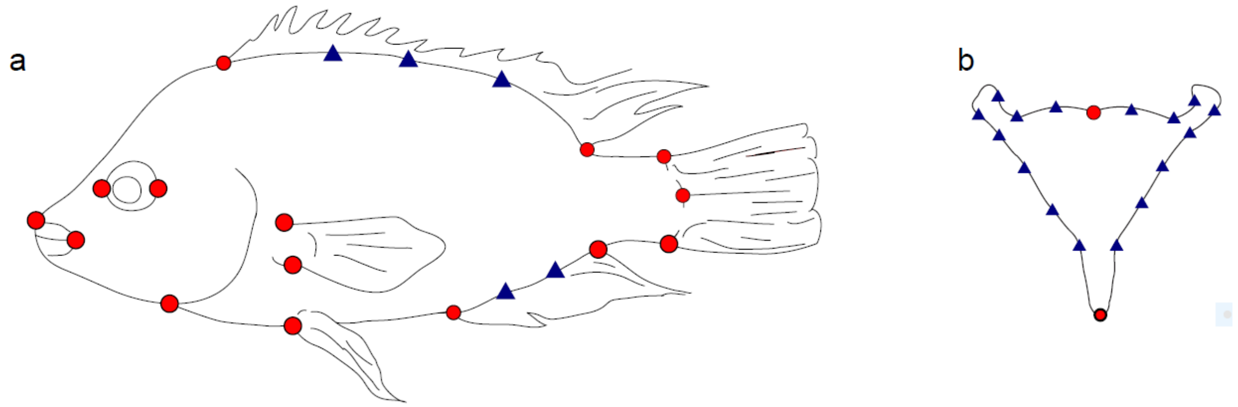

Supplementary Figure 3 | Configuration of points used in the geometric morphometric analyses of shape. Points in red have been treated as landmarks, blue triangles represent semilandmarks. (**a**) Body. (**b**) Lower pharyngeal jaw.

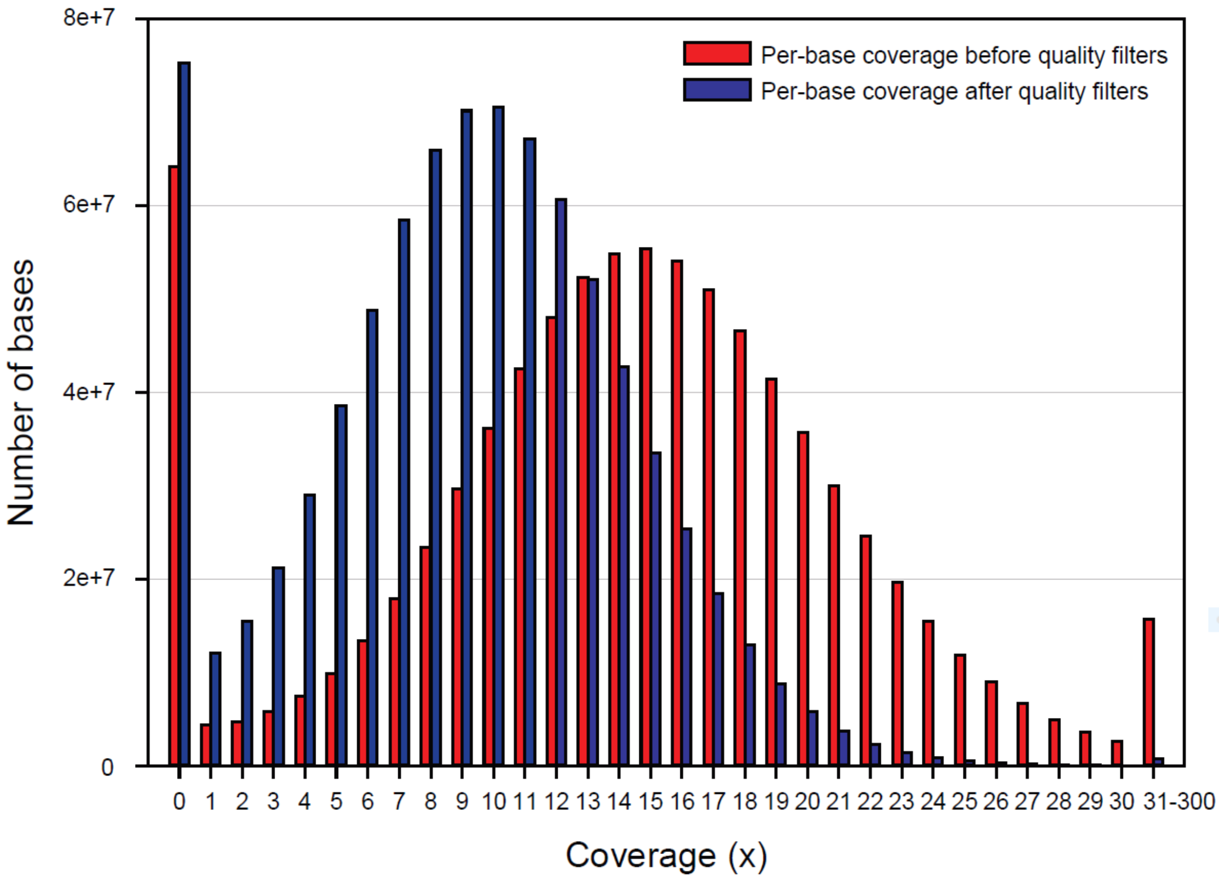

Supplementary Figure 4 | Coverage distribution of the PoolSeq dataset. The bar plot shows the per-base coverage distribution along the Midas genome of the two species pools combined. The grouped red and blue bars indicate the coverage before and after the quality filters applied (see Methods for details).

Supplementary Tables

Trait	LG	LOD score	Bayesian credible interval (cM)	Additive effect (SS)	Additive effect (%)	Additive effect (LOD)	Dominant effect (SS)	Dominant effect (%)	Dominant effect (LOD)
Body shape	3	5.8184	18-23-28	0.0047	3.86	10.83	0.0024	1.96	0.07
Body shape	5	3.6775	0-3-17	0.0019	1.52	5.9	0.0023	1.91	0.27
Body shape	6	3.3903	8-17.4-23.2	0.0025	2	2.95	0.0026	2.1	1.35
Body shape	9	3.7923	4-16.8-23	0.0038	3.07	5.43	0.0029	2.33	0.47
Body shape	19	3.4841	38-47-56	0.002	1.64	4.73	0.002	1.62	0.48
Pharyngeal jaw shape	3	3.7369	6-11-24	0.0048	4.08	3.19	0.0035	3	1.51
Pharyngeal jaw shape	12	3.9260	24-33-54	0.0034	2.92	2.78	0.0064	5.47	2.03
Pharyngeal jaw shape	15	4.3561	9-11-18	0.0031	2.62	3.77	0.0026	2.2	1.61
Shape covariation	3	5.5507	16-22-31	0.0064	8.56	6.81	0.0011	1.5	0.14

Supplementary Table 1 | Position and effect of QTL of body shape, pharyngeal jaw shape and their covariation. Summary of the QTL significant at the permutational genome-wide threshold. LOD scores are provided for the peak at the center of the Bayesian credible interval. Permutational-derived LOD significance thresholds are 3.39 for body shape, 3.56 for pharyngeal jaw shape and 3.52 for their covariation. The Bayesian credible interval is provided in centiMorgan in the form “lower extreme-center-upper extreme”. QTL effects are expressed as sum of squares and as percentage of the total Procrustes sum of squares in the mapping population.
